# Women's attitude towards intimate partner violence and utilization of contraceptive methods and maternal health care services: an analysis of nationally representative cross-sectional surveys from four South Asian countries

**DOI:** 10.1186/s12905-022-01780-4

**Published:** 2022-06-08

**Authors:** Som Kumar Shrestha, Subash Thapa, Don Vicendese, Bircan Erbas

**Affiliations:** 1 Save the Children Nepal/Global Fund, Kathmandu, Nepal; 2 Department of Medical Statistics, London School of Hygiene and Tropical Medicine, London, UK; 3 Research Unit of General Practice, Department of Public Health, University of Southern Denmark, Odense, Denmark; 4 Department of Mathematics and Statistics, La Trobe University, Melbourne, Australia; 5 Melbourne School of Population and Global Health, University of Melbourne, Melbourne, Australia; 6 School of Psychology and Public Health, La Trobe University, Melbourne, Australia

**Keywords:** Intimate partner violence, Contraceptive methods, Antenatal care, Institutional delivery and post-natal care

## Abstract

**Background:**

Intimate partner violence (IPV) adversely affects women’s reproductive health outcomes but to what extent women’s justification of IPV affects maternal health care service utilization is unexplored.

**Methods:**

The secondary cross-sectional datasets from multiple indicator cluster surveys of Afghanistan, Bhutan, Nepal and Pakistan conducted between 2010 and 2015 were used. We used a generalized linear mixed model with random effects, at both cluster- and country-level, to determine the odds ratio of maternal health service utilization at the regional level and a multivariable logistic regression model adjusting for complex survey design at the country level. Interaction between women’s justification of IPV and residential location, and linear trend in the utilization of maternal health care services associated with increasing levels of women's justification of IPV, were examined using the Likelihood Ratio Test (LRT).

**Results:**

A total of 26,029 women aged 15–49 years, living with their partners and had a pregnancy outcome 2 years prior to the survey were included. Women justifying IPV were less likely to utilize contraceptive methods (aOR) = 0.86, 95% CI 0.84, 0.88), at-least one Antenatal Care (ANC) visit (aOR = 0.80, 95% CI 0.72, 0.88), four or more ANC services (aOR = 0.81, 95% CI 0.76, 0.86), institutional delivery (aOR = 0.87, 95% CI 0.80, 0.94) and Post-natal Care (aOR = 0.76, 95% CI 0.62, 0.95) services. A decreasing linear trend was observed for four or more ANC visits (LRT *P* = 0.96) and institutional delivery (LRT *P* = 0.80) with increasing levels of IPV justification. Women justifying IPV were less likely to have at least one ANC visit in urban (aOR 0.67, 95% CI 0.60, 0.75) compared to rural areas (aOR 0.83, 95% CI 0.73, 0.94).

**Conclusions:**

Women’s justification of IPV was associated with decreased odds of utilizing a wide range of maternal health care services at the regional level. Although further research that may help establish a causal link is important before formulating public health interventions, our study indicates interventions targeting women’s condoning attitude toward IPV, delivered sooner rather than later, could potentially help to improve women’s utilization of essential maternal health care services in the South Asian region that comprises Afghanistan, Bhutan, Nepal, and Pakistan.

**Supplementary Information:**

The online version contains supplementary material available at 10.1186/s12905-022-01780-4.

## Background

According to the World Health Organization (WHO), Intimate Partner Violence (IPV) is any behaviour within an intimate relationship that causes physical, psychological or sexual harm to those in the relationship, including acts of physical aggression, sexual coercion, psychological abuse, and controlling behaviour [1]. IPV is the most common form of violence against women, with global estimates suggesting 29% of women aged 15 to 49 years experienced some sort of physical and/or sexual violence by their intimate partners at least once in their lifetime [2, 3], and the prevalence varies widely between countries and regions [2]. Women from South Asian countries face a high burden of IPV with 51% of women in Afghanistan [4], 39% in Pakistan [5], 26% in Nepal [6] and 27% in Bhutan [7] having reported experiencing either physical or sexual violence by their partners at least once in their life. This high prevalence of IPV among South Asian countries has major health, social and economic consequences for women, families and the government [8].

IPV is a complex phenomenon that can occur in all settings and affects women disproportionally [9]. IPV is highly influenced by local-contextual forces that vary among and within countries, and there are risk factors for experiencing IPV that can be consistently identified across different settings [9]. For instance, women who are unemployed and have a low level of education are likely to be exposed to violence between partners [9, 10]. In addition to these individual factors, structural and social factors, such as patriarchal societal structure limiting economic opportunities for women, conflict situations, poverty, social norms, gender inequality and weak legal frameworks for women’s civil rights are also identified as potential risk factors for perpetration of IPV [1, 11]. The South Asian region has deep rooted social and patriarchal norms that restrict women’s freedom and expects them to behave in particular ways which are identified as critical drivers of IPV. The policy and legal frameworks for preventing and tackling IPV vary considerably across different countries in South Asia but the gap in implementation and not giving adequate attention to underlying social norms and values that drive IPV has often hindered progress to achieve tangible outcomes and to reduce the burden of IPV in the region [12].

A growing number of socio-epidemiological studies have consistently reported negative consequences of IPV on women’s health [10, 13]. Studies have particularly highlighted important associations between the incidence of IPV and adverse maternal health outcomes such as miscarriage, stillbirth, labour complications and other pregnancy or delivery complications [14, 15]. One of the pathways between incidence of IPV and poor maternal health outcomes is due to reduced likelihood of utilizing essential reproductive healthcare services during pregnancy, childbirth and other lifetime events [13, 15]. In a society where inequalities between men and women is high in terms of access to resources and decision making, it substantially increases the risk of IPV and the women may further feel powerless and fear abusive experiences from their partners [16]. In this context, women are often reluctant to access healthcare services without the husband’s approval, which could act as a barrier to utilize essential reproductive health services consequently leading to adverse health outcomes [10].

Despite the abundance of studies linking violence incidence with poor maternal health outcomes [14, 15], little is known about whether women’s justification of IPV affect their ability to utilize essential maternal health care services. Women’s condoning attitude towards IPV that depict women’s justification of violence from their partner could be portrayed as one of the barriers for accessing maternal health care services. As most interventions have been mainly focused on prevention strategies to address the incidence of IPV in South Asia [17], evidence linking women’s justification of IPV and maternal health care service utilization could provide new perspectives to tackle community attitude towards violence in an effort to improve women’s access to essential maternal health care services.

To the best of our knowledge, no prior study has investigated the association between women’s justification of IPV and utilization of the range of maternal health care services in a comprehensive manner that are essential for women to fully utilize their reproductive health rights. In this study, we aim to fill this gap through examining the association between women’s justification of IPV and use of contraceptive methods, ANC services, institutional delivery, and post-natal care (PNC) services using nationally representative samples from the South Asian region. To be specific, the study has the following objectives: 1) To examine the association between women’s justification of IPV and contraceptive methods and maternal health care service utilization in four South Asian countries; 2) To investigate interaction between women’s justification of IPV and area of residence on contraceptive use and maternal health care services; 3)To examine if there is a linear association between increasing levels of women’s justification of IPV and contraceptive and maternal health care service utilization; and 4) To examine if women’s justification of IPV is associated with delay in accessing the first ANC visit. The evidence could be used to formulate appropriate public health policies and programmes to tackle community acceptance of IPV as a part of broader strategies to improve women’s reproductive health needs in the South Asian countries.

## Methods

### Study context

The study included four South-Asian countries Afghanistan, Bhutan, Nepal, and Pakistan (Punjab and Sindh provinces). All countries belonged to lower or lower-middle-income countries with high incidences of poverty and social and health inequalities [18]. Among the countries selected in this study, poverty headcount ratio at national poverty lines (percentage of population) was the highest for Afghanistan (54.5%) and the lowest for Bhutan (8.2%). The estimated total population was 37.17 million in Afghanistan, 0.75 million in Bhutan, 28.09 million in Nepal and 212.22 million in Pakistan in 2018 [19]. South Asian nations have low health standard compared to other regions [18]. The life expectancy at birth was the lowest for Afghanistan with 64 years, followed by 67.11 years for Pakistan, 70.48 years for Nepal and 71.46 years for Bhutan [19].

### Data source and sampling

The study used data from the Multiple Indicator Cluster Survey (MICS) from four South-Asian countries; namely Afghanistan, 2010–11; Bhutan, 2010; Nepal, 2014; and Pakistan (Punjab, 2014 and Sindh, 2014). The MICS is based on nationally representative samples from Afghanistan, Bhutan and Nepal; however, Pakistan consists of two independent studies from Punjab and Sindh provinces. The surveys were based on a cross-sectional study design and multi-stage sampling methods [20]. At first, enumeration areas (EA) were selected systematically with probability proportional to their size and the required number of samples was selected from each EA in the second stage. Methodology and sampling design details are described elsewhere [4, 21–24]. A total of 95,616 households were included in this study with an overall response rate of 97.4%, with 129,785 women of reproductive age (15–45 years) being interviewed which accounted for an 89.5% response rate. A total of 26,029 women who were either married or living together with their partners and had a pregnancy outcome (live births) 2 years prior to the survey were included in this study (Additional file 1: Table S1).

### Outcome variables

The outcome variables included current utilization of contraceptive methods, at-least one ANC visit, completed four or more ANC visits, institutional delivery and PNC services. The contraceptive methods included both traditional and modern methods. We included both having at-least one ANC visit and four or more ANC visits as the outcome variables. The childbirth at government hospitals, primary health care center, private hospitals, private clinics or health institutions managed by non-governmental organizations (NGOs) were included as an institutional delivery. The PNC indicator included women who had their health check-up within two days of the most recent birth, either at home or at health institutions. The PNC visit information is only available for Nepal and Pakistan.

### Exposure variables

IPV usually denotes physical, sexual, and emotional abuse and controlling behaviours by their intimate partners [1]. For the purpose of this study, women’s justification of IPV denotes a condoning attitude towards physical violence perpetrated by their partners. The exposure variable, women’s justification of IPV, was measured through standard tools used by MICS and Demographic Health Surveys (DHS) [6, 20]. It was based on a series of questionnaires that collected information on women’s attitudes towards wife-beating by their husband/partner under different conditions. Women were asked if wife-beating is justified for different conditions. They were specifically asked if wife-beating is justified for going out without informing their husband, neglecting children, arguing with their husband, refusing to have sex with their husband and burning food. A dichotomous variable was created to document women’s justification of IPV if they justified wife-beating for any one of the conditions presented to them.

The variable capturing the levels of women’s justification of IPV was generated to measure the extent to which women justified the conditions for wife-beating, ranging from 0 (not justifying any option) to 5 (justifying all five options). The definition of all outcomes, exposure and other covariates used in the study are provided in Additional file 1: Table S2.

### Statistical analysis

The datasets from all four selected countries were merged for the purpose of analysis. The combined dataset constitutes a hierarchical structure with more than one level of clustering, for example in the combined dataset, households are clustered within primary sampling units (i.e. sampling clusters), sampled clusters within countries and countries within a regional level. Therefore, modelling of the outcome variables should take into account the correlations within clusters that vary between them [25].

The study used a generalized linear mixed model with random effects at both cluster and country level with the households nested within the country. The detail description of the methods is provided elsewhere [26] but a brief description of the multi-level model is provided below.

Let, ${y}_{i\left(j\right)t}$ denote the response for woman *t *who lives in cluster *i* and country *j*, with 1 = occurrence of event/outcome and 0 = No occurrence of event for the outcome variables. Then, a multilevel model with random effect {${v}_{i\left( j \right)}\}$ for clusters and ${\{u}_{\left( j \right)}\}$ for country and fixed effects for explanatory variables is given by the following equation (27).

($${\text{Logit }}[{\text{P(}}y_{i\left( j \right)t} ) = {1})] \, = {\varvec{x}}_{{{\varvec{i}}\left( {\varvec{j}} \right){\varvec{t}}}}^{{\varvec{T}}} { }\beta + u_{{\left( {{ }j{ }} \right)}} + { }v_{{i\left( {{ }j{ }} \right)}}$$.

where ${{\varvec{x}}}^{{\varvec{T}}}$ represents the vector of explanatory variables for women *t* living in cluster *i* and country *j*. $\beta$ represents the fixed effect parameter that have conditional interpretations given the random effect. ${v}_{i\left( j\right)}$ denotes level-1 (cluster level) random effects that account for variability among respondents, i.e. women, within a cluster. ${u}_{\left( j\right)}$ denotes level-2 random effects that accounts for variability among countries. The random components ${u}_{\left( j\right)}$ and ${v}_{i\left( j\right)}$ are assumed to be independent with distributions N (0, ${\sigma }_{u}^{2}$) and N (0, ${\sigma }_{v}^{2}$) respectively.

We calculated the aggregated effect size estimates of contraceptive and maternal health care service utilization associated with women’s justification of IPV at the regional level. The country-level analysis was performed using a multivariable logistic regression model. The model was also used to analyse differences in the time (months) for the first contact with healthcare facilities after pregnancy associated with women’s justification of IPV. A likelihood ratio test was used to evaluate a linear trend in utilization of contraceptive methods and maternal health care services associated with increasing levels of women’s justification of IPV. We also checked for interaction between women’s justification of IPV and the area of residence on the outcome variables.

We controlled for a wide range of confounding variables in the models that were identified in the existing literature [28–30]. Variables such as women’s age, women’s education status, area of residence and household wealth quintiles were entered in the model as categorical variables. Likewise, women’s age at first marriage/union, age of husband and number of children ever born were entered in the model as continuous variables.

Sampling weights for women were used to adjust for the complex survey sampling design and non-proportionate selection probability in the analysis. We adjusted for the country level weights in addition to the women weights for pooling the results at the regional level [31]. The *P* values are 2-sided and statistical significance level set at less than 0.05. This study used Stata 14.1 (Stata Corp, College Station, Texas) for data analysis.

## Results

### Demographic characteristics

Table 1 shows the socio-demographic characteristics of the women included in the study. There is a wide variation in the age groups of women included in the study across different countries (*P* < 0.001). Nearly, 10.5% of women were aged between 15–19 years in Nepal, while that age group represented only 3.3% and 4.9% of the total sample population for Punjab and Sindh province of Pakistan respectively (Table 1).

**Table 1:** Socio-demographic characteristics of reproductive age women by country

	Afghanistan (N = 4865) (%)	Bhutan (N = 2368) (%)	Nepal (N = 2048) (%)	Pakistan (Punjab) (N = 10,653) (%)	Pakistan (Sindh) (N = 6095) (%)	Chi-square test statistics	*P* value
Women’s Age							
15–19	8.3	5.9	10.5	3.3	4.9	830	< 0.001
20–24	27.4	30.3	35.0	20.5	23.4		
25–29	29.6	33.0	33.0	35.2	33.6		
30–34	16.0	18.0	13.5	25.1	22.8		
35–39	12.2	8.8	5.3	11.7	10.2		
40–44	4.6	3.1	2.1	3.4	3.9		
45–49	1.9	0.9	0.6	0.8	1.2		
Mean age (years) of husband (± SD)	42.7 [24.0]	32.1 [11.3]	30.4 [10.5]	33.9 [9.9]	36.2 [16.4]	-	< 0.001*
Mean women’s age (years) at first marriage (± SD)	17.7 [6.8]	18.9 [3.8]	17.7 [4.0]	20.2 [4.5]	18.7 [4.5]	-	< 0.001*
Education							
None	88.6	62.7	36.8	45.2	55.4	2900	< 0.001
Primary	5.9	12.8	16.9	18.4	15.2		
Secondary or higher	5.5	24.6	46.3	36.4	29.4		
Area							
Rural	18.6	29.2	12.8	30.8	46.1	575	< 0.001
Urban	81.4	70.9	87.2	69.2	53.9		
Wealth quintiles							
Poorest	19.2	19.9	22.2	21.8	24.8	1100	< 0.001
Second	21.1	18.9	21.3	20.3	22.2		
Middle	20.4	20.0	21.6	20.1	20.7		
Fourth	19.9	21.9	19.6	19.4	17.1		
Richest	19.4	19.2	15.4	18.3	15.2		

Surveys countries and year: Afghanistan 2010–11; Bhutan 2010; Nepal 2014; Pakistan (Punjab), 2014 and Pakistan (Sindh), 2014

^*^P value from one-way ANOVA test

### IPV prevalence

The proportion of women justifying IPV for different conditions, across different countries is presented in Table 2. In general, 58.8% of the women agreed that IPV is justified for at least one reason but the prevalence varied widely across different countries (Table 2).

**Table 2:** Women’s justification of intimate partner violence by country

Justified wife- beating	Afghanistan (N = 4865) n (%)	Bhutan (N = 2368) n (%)	Nepal (N = 2048) n (%)	Pakistan Punjab (N = 10,653) n (%)	Pakistan Sindh (N = 6095) n (%)	Total (N = 26,029) n (%)
Goes out without telling the husband	3983 (81.9)	899 (38.0)	576 (28.1)	3217 (30.2)	2663 (43.7)	11,337 (43.6)
Neglects the children	3148 (64.7)	1286 (54.3)	711 (34.7)	3188 (29.9)	2667 (43.8)	11,001 (43.3)
Argues with the husband	3904 (80.3)	905 (38.2)	383 (18.7)	3338 (31.3)	2657 (43.6)	11,186 (43.0)
Refuses to have sex with the husband	2614 (53.7)	607 (25.7)	64 (3.1)	2782 (26.1)	2458 (40.3)	8526 (32.8)
Burns food	1689 (34.7)	569 (24.1)	109 (5.3)	1781 (16.7)	2148 (35.2)	6297 (24.2)
At-least one of the options	4574 (94.0)	1625 (68.6)	944 (46.1)	4705 (44.2)	3452 (56.6)	15,300 (58.8)

Surveys countries and year: Afghanistan 2010–11; Bhutan 2010; Nepal 2014; Pakistan (Punjab), 2014 and Pakistan (Sindh), 2014

### Prevalence of contraceptive methods and maternal health care service utilization

Contraceptive methods and maternal health care service utilization pattern among women who had a pregnancy over the last 2 years is presented in Table 3. In general, 33.4% of the women were using contraceptive methods, 78.9% of the women had at-least one ANC visit and 43.7% had four or more ANC visits and 56.1% had institutional delivery, while overall 77.9% of the women had PNC services (data available from Nepal and Pakistan only) (Table 3).

**Table 3:** Contraceptive methods use and maternal health care service utilization among reproductive-aged women by country

Countries	Contraceptive methods N (%)	At least one Antenatal Care visit N (%)	Four or more Antenatal Care visits N (%)	Institutional Delivery N (%)	Post Natal Care services N (%)
Afghanistan (n = 4865)	1030 (21.2)	2688 (55.3)	711 (14.6)	1600 (32.9)	n/a
Bhutan (n = 2368)	1468 (62.0)	2309 (97.5)	1831 (77.3)	1495 (63.1)	n/a
Nepal (n = 2048)	620 (30.3)	1776 (86.7)	1218 (59.5)	1130 (55.2)	1168 (57.0)
Pakistan Punjab (n = 10,653)	3880 (36.4)	8815 (82.8)	5118 (48.1)	6473 (60.8)	9163 (86.0)
Pakistan Sindh (n = 6095)	1686 (27.7)	4949 (81.2)	2506 (41.1)	3901 (64.0)	4320 (70.9)
Total (n = 26,029)	8684 (33.4%)	20,538 (78.9)	11,384 (43.7)	14,598 (56.1)	14,651 (77.9) *

Surveys countries and year: Afghanistan 2010–11; Bhutan 2010; Nepal 2014; Pakistan (Punjab), 2014 and Pakistan (Sindh), 2014

*Proportion calculated based on total women population of 18,796 as the denominator

### Association between women’s justification of IPV and maternal reproductive health care service utilization

The unadjusted and adjusted odds ratios of use of contraceptive methods and maternal health care services associated with women’s justification of IPV are provided in Table 4. In general, the unadjusted odds ratio of using contraceptive methods and maternal health care services decreased substantially with women’s justification of IPV at the regional level (Table 4).

**Table 4:** The odds ratio of contraceptive methods and maternal health care service utilization associated with women’s justification of intimate partner violence

Country	Contraceptive methods		At least one Antenatal Care visit		Four or more Antenatal Care visits		Institutional delivery		Post-natal care services	
	Unadjusted	Adjusted^♱^	Unadjusted	Adjusted^♱^	Unadjusted	Adjusted^♱^	Unadjusted	Adjusted^♱^	Unadjusted	Adjusted^♱^
Afghanistan	0.72 (0.54, 0.93)	1.02 (0.76, 1.36)	0.59 (0.43, 0.80)	0.86 (0.63, 1.16)	0.39 (0.28, 0.53)	0.62 (0.46, 0.84)	0.51 (0.37, 0.69)	0.88 (0.65, 1.19)	N/A	N/A
Bhutan	1.07 (0.86, 1.32)	1.14 (0.90, 1.43)	1.56 (0.93, 2.60)	1.66 (0.91, 3.03)	0.97 (0.76, 1.24)	1.13 (0.87, 1.47)	0.77 (0.61, 0.97)	0.95 (0.73, 1.25)	N/A	N/A
Nepal	0.83 (0.66, 1.06)	0.91 (0.71, 1.15)	0.51 (0.36, 0.72)	0.64 (0.45, 0.92)	0.62 (0.50, 0.77)	0.85 (0.68, 1.07)	0.53 (0.42, 0.68)	0.72 (0.55, 0.94)	0.48 (0.38, 0.60)	0.62 (0.48, 0.79)
Pakistan Punjab	0.73 (0.66, 0.80)	0.86 (0.78, 0.95)	0.55 (0.48, 0.62)	0.88 (0.77, 0.99)	0.49 (0.44, 0.54)	0.84 (0.76, 0.93)	0.55 (0.50, 0.61)	0.92 (0.83, 1.01)	0.67 (0.59, 0.76)	0.85 (0.74, 0.97)
Pakistan Sindh	0.48 (0.41, 0.57)	0.93 (0.76, 1.13)	0.40 (0.33, 0.48)	1.02 (0.84, 1.25)	0.29 (0.25, 0.34)	0.80 (0.68, 0.94)	0.39 (0.33, 0.45)	0.94 (0.81, 1.11)	0.58 (0.49, 0.68)	1.18 (0.98, 1.41)
All countries combined*	0.74 (0.66, 0.81)	0.86 (0.84, 0.88)	0.53 (0.50, 0.57)	0.80 (0.72, 0.88)	0.52 (0.46, 0.59)	0.81 (0.76, 0.86)	0.57 (0.54, 0.60)	0.87 (0.80, 0.94)	0.57 (0.48, 0.68)	0.76 (0.62, 0.95)

Surveys countries and year: Afghanistan 2010–11; Bhutan 2010; Nepal 2014; Pakistan (Punjab), 2014 and Pakistan (Sindh), 2014

^♱^Models adjusted for women’s age, women’s education, area of residence, women’s age at first marriage/union, age of husband, wealth quintiles, and number of children ever born

*Adjusted for both cluster level women weights and country weights in the model

In the adjusted model, women who justified IPV for at least one reason were significantly less likely to use contraceptive methods (aOR = 0.86, 95% CI  0.84, 0.88), ANC visit (aOR = 0.80, 95% CI 0.72, 0.88), four or more ANC visits (aOR = 0.81, 95% CI 0.76, 0.86), institutional delivery (aOR = 0.87, 95% CI 0.80, 0.94) and PNC services (aOR = 0.76, 95% CI 0.62, 0.95) at the regional level (Table 4).

There was evidence of interaction between area of residence and women’s justification of IPV for having at least one ANC visit (Interaction Term aOR: 0.81 (95% CI 0.93, 0.70); *P* = 0.003), where the odds of accessing ANC service associated with women’s justified IPV was lower in urban (aOR 0.67, 95% CI 0.60, 0.75) area compared to the women from the rural area (aOR 0.83, 95% CI 0.73, 0.94) (Additional file 1: Table S3). However, no such effect was observed for contraceptive methods use, four or more ANC visits, institutional delivery and PNC visit (result not shown).

**Fig. 1:**
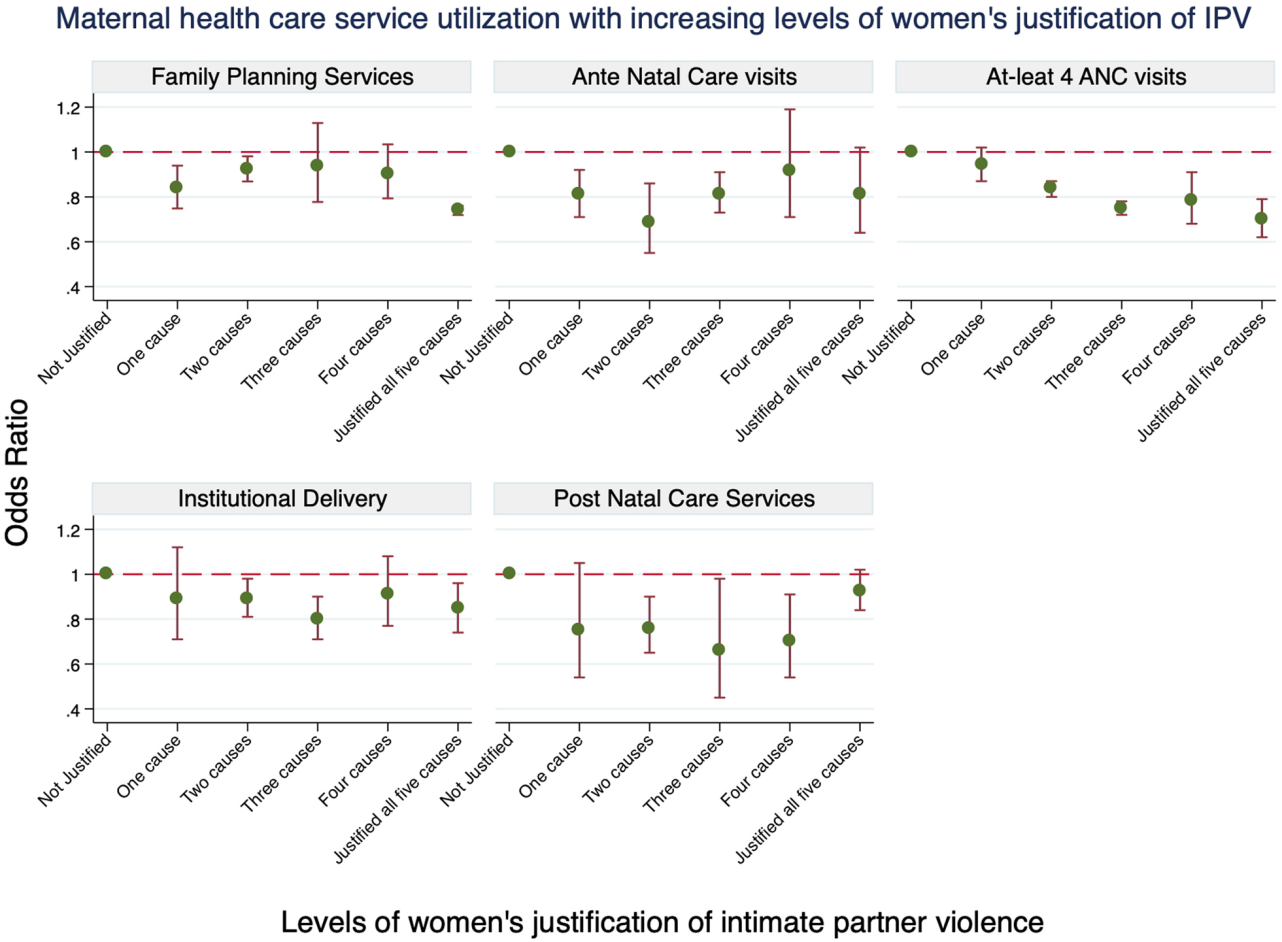
Contraceptive methods and maternal health care service utilization trend with increasing levels of women’s justification of IPV

The study found a linear decreasing trend of utilization of four or more ANC visits (LRT chi^2^ = 0.62, *df* = 4, LRT *P* value = 0.96) and institutional delivery (LR chi^2^ = 1.67, *df* = 4, LRT *P* value = 0.80) with increasing levels of women’s justification of IPV. However, the association was less obvious for contraceptive methods and ANC services with a non-linear pattern observed across different levels of justification of violence (Fig. 1), but still, the statistical tests suggest no evidence against the linear association, LR chi^2^ = 2.83, *df* = 4, LRT *P* value = 0.59 and LR chi^2^ = 6.06, *df* = 4, LRT *P* value = 0.20 respectively. There was some weak evidence against a linear trend assumption for PNC visit outcome (LR chi^2^ = 8.94, *df* = 4, LRT *P* value = 0.06) (Fig. 1).

There was some evidence of delay in accessing the first ANC visit associated with women’s justification of IPV in Pakistan but no association was observed in Nepal. Overall, women who justified IPV delayed accessing first ANC visit by an average of 0.22 months (95% CI 0.13, 0.31) in Punjab and 0.36 months (95% CI 0.22, 0.49) in Sindh provinces of Pakistan respectively compared to the women who did not justify IPV (Additional file 1: Table S4). The population average marginal means of first ANC visits among women who did not justify IPV was 2.86 months for Punjab Pakistan (95% CI 2.79, 2.92) and 3.71 months for Sindh Pakistan (95% CI 3.59, 3.83).

## Discussion

This study found a significant inverse association between women’s justification of violence by their partner and the utilization of contraceptive methods and maternal health care services in the South Asian countries. We also observed a linear decreasing trend of utilizing four or more ANC visits and institutional delivery associated with the increasing levels of women’s justification of IPV. The study contributes to the existing literature in understanding how women’s justification of violence from their partner could limit their ability to access contraceptives and maternal health care services.

In the present study, overall, more than half (58.8%) of the women reported that IPV was justified for at least one of the conditions presented to them, showing a fairly high level of women’s approval of violence by their intimate partners in South Asia. However, prevalence varied widely across different countries. It ranged from 44.2% in Punjab Pakistan, the lowest, to 94% in Afghanistan, the highest proportion in the region. In comparison to all other countries, women from Afghanistan consistently reported higher approval of IPV. The higher proportion of women justifying violence in Afghanistan might be related to the lower socio-economic status of women in the society, and the long period of conflict and civil war endured by the country that is known to increase women’s vulnerability and the risk of exploitation and violence [32]. The prevalence of utilization of maternal health care services also varied widely across different countries. Afghanistan had the lowest utilization for all maternal health care services, whereas Bhutan had the highest prevalence of family planning services, at least one ANC visit and four or more ANC visits among the four nations. Relatively high uptake of maternal health care services including ANC services in Bhutan could be attributed to the free health care service policy and priority set by the government to improve maternal and child health status in recent years [33]. These outcomes illustrate contextual differences across the South Asian countries in the distribution of women’s attitudes towards IPV and access to maternal health care services and hence the need to adjust for these differences with a multi-level analysis strategy as done in this study.

Although previous studies have shown important links between the experience of violence and negative health consequences [29, 34], this is the first study that provided direct evidence linking women’s justification of IPV and lower utilization of wide range of reproductive healthcare services. We compared our findings with other studies that have examined the association between incidence of violence and reproductive health service utilization. Our findings are similar to other studies conducted in South Asia that have reported a lower likelihood of maternal health service utilization associated with women’s experience of IPV [29, 34, 35].

The multilevel modelling showed that the use of contraceptive methods was lower among women who justified IPV but another pooled analysis of the countries from the South Asian Region found that, women who had experienced violence from partners, were more likely to use modern contraceptive methods [30]. The contradictory findings could be partly explained due to methodological differences, where women’s justification of IPV could be much different to actual experience of violence. Nevertheless, our study suggests Family planning (FP) policies targeting vulnerable women who justify IPV is likely to benefit the large number of reproductive aged women living in South Asian countries to prevent unwanted births, need for abortion and pregnancy-related complications [36].

Women’s justification of IPV was strongly associated with lower utilization of ANC visits which is consistent with other studies from the South Asian region [29, 34]. Furthermore, our study also showed that women who justified IPV were more likely to delay first contact with health facilities to access the ANC visit after pregnancy in Pakistan, but the association was not statistically significant in Nepal. Though the actual reason for such delay is unclear, a similar pattern was also observed from another study that related women’s incidence of violence with the timing of prenatal care [37]. Timing of the first ANC visit is important for early detection, management and prevention of complications that could occur during pregnancy [38]. Our study suggests that women who justified violence delayed having the the first ANC visit by an average of almost 7 days in the Pakistan Punjab and 11 days in the Pakistan Sindh provinces compared to their counterparts. Delay in contacting health facilities after pregnancy also means women lag behind in receiving essential interventions, delivered through health facilities, for preventing pregnancy complications and improving the health of mothers and unborn child [39].

Even more concerning is that women who justified at-least one form of IPV had an increased risk of not delivering their child at healthcare facilities and were even less likely to access PNC services after childbirth. WHO recommends that every delivery should be conducted by skilled health professionals within a well-functioning healthcare system that can manage several complications of pregnancy and childbirth [40]. Women’s justified IPV was found to be a major barrier for receiving essential care during pregnancy and childbirth which is critical to both women’s and child’s health and their survival.

Our study reported evidence of interaction between the women’s justification of IPV and area of residence for having at least one ANC visit. The adverse association between women’s justified IPV and ANC visit was stronger in urban areas compared to rural areas. Women from urban locations have a relatively greater access to ANC services compared to rural locations [41, 42], probably due to better treatment options, availability of services, and easy access to transportation. However, our study showed that women’s justification of violence amplifies the barriers to access ANC services in urban settings, but the relationship could have been mediated through other economic and social determinants and further studies could elucidate possible causes for such differences.

It is also important to note that our observed association between women’s perceived justification of violence and utilization of maternal health care services varied widely across different countries. The significant negative association for utilization of contraceptive methods was observed only for the Punjab province of Pakistan, likewise the adverse association for institutional delivery was only significant for Nepal. Our findings illustrate the need for considering residential location and country-specific patterns to respond to current disparities in the utilization of reproductive healthcare services associated with women’s perceived justification of violence across different countries.

Women’s attitude towards intimate partner violence is often driven by deeply rooted gender norms and beliefs within patriarchal power structures that promote male dominance and condone erosion of women’s rights [43]. Studies have shown women's justification of IPV as a risk factor for women's experience of violence [44]. Several multi-sectoral and systemic approaches have been identified as possible interventions to prevent incidence of IPV in a South Asian context [12, 17]. Strengthening legal frameworks and social measures to support gender equality and women’s rights, investment in women’s education and economic empowerment and engaging with local communities to shift harmful gender attitudes and norms are a few examples of interventions that could be scaled-up at national and community levels [12, 17, 45].

Our study findings are based on large nationally representative samples from South Asia except for Pakistan and could be generalized to most of the countries in the region with similar settings. The country-level samples included in this study were based on the MICS survey which provided a consistent survey methodology and sampling design for ensuring comparability and generalization of results. We presented both the country specific and the regional level outcome measures to inform policy makers and planners to evaluate evidence at different levels. We believe our choice of indicator, women’s justification of IPV rather than actual violence incidence is more appropriate to mitigate bias associated with capturing sensitive information like violence incidence in cross-sectional studies [46, 47].

This study has some limitations. Though most of the samples were nationally representative, the samples from Pakistan were based on Punjab and Sindh provinces solely but these two provinces represented nearly 76% of the total population and therefore would most likely fairly represent the population at the national level [48]. Differential non-response could bias the findings but with our high response rate, over 85% in all countries, it might not have substantially altered interpretation of the results. We did a sensitivity analysis taking 8 or more ANC visits as the outcome considering recent revised guidelines from WHO [49]. The odds ratio of completing at-least eight ANC visits among those who justified IPV was even lower (aOR: 0.76, 95% CI 0.64, 0.91) (Additional file 1: Table S5) compared to four or more ANC visits (Table 4). Furthermore, the surveys in different countries were conducted at different time points which could limit the comparability of results, but all studies were conducted between 2010 and 2014, so comparability of survey findings might not be a major issue. Despite, controlling for important confounders, we could not fully ignore the effect of residual confounding. Other factors such as accessibility and proximity of services, and quality of service delivered by health facilities, could also have influenced utilization of reproductive healthcare services.

## Conclusion

Women’s justification of IPV was a strong determinant of the use of contraceptive methods, first ANC visit, 4 or more ANC visits, institutional delivery, and PNC services in the South Asian countries. However, the pattern and significance of associations for specific health outcomes varied across different countries. These findings have important policy implications. Our findings particularly highlighted the important link between women’s condoning attitude towards IPV and lower utilization of maternal and reproductive health services. Though further studies might be essential to establish a causal link, public health interventions delivered sooner rather than later, that focus on women’s empowerment and education in partnership with men’s education about their role within an intimate relationship could potentially benefit to improve women’s access to reproductive health care services in the region. The study also suggests considering the country-specific context and rural–urban differences while formulating interventions targeting specific reproductive health outcomes for women in the South Asian region.

## Supplementary Information

**Additional file 1:** **Table S1**. A summary of total households and women samples included in the study. **Table S2** Definition of outcome, exposure, and other covariates included in the study. **Table S3** Adjusted odds ratio for at least one Antenatal care visit associated with women’s justification of intimate partner violence by area of residence and its interaction. **Table S4** Delay in timing (month) for the first Antenatal care visit associated with women’s justification of intimate partner violence. **Table S5** Unadjusted and adjusted odds ratio of women having 8 or more Antenatal care visits associated with women’s justification of intimate partner violence.

## Data Availability

All datasets used in the currently study are available from Multiple Indicator Cluster Survey website, https://mics.unicef.org/surveys on request.

## Abbreviations

ANC: Antenatal care
CI: Confidence interval
EA: Enumeration area
IPV: Intimate partner violence
MICS: Multiple indicator cluster surveys
PNC: Post natal care
PSU: Primary sampling units
WHO: World Health Organization
