# Supplementary material for: Women's attitude towards intimate partner violence and utilization of contraceptive methods and maternal health care services: an analysis of nationally representative cross-sectional surveys from four South Asian countries
Source: BMC Womens Health. 2022 Jun 8;22:215. doi: 10.1186/s12905-022-01780-4 (PMC9178873; doi:10.1186/s12905-022-01780-4)
Supplement: Supplementary file 1 — Additional file 1: Table S1. A summary of total households and women samples included in the study. Table S2 Definition of outcome, exposure, and other covariates included in the study. Table S3 Adjusted odds ratio for at least one Antenatal care visit associated with women’s justification of intimate partner violence by area of residence and its interaction. Table S4 Delay in timing (month) for the first Antenatal care visit associated with women’s justification of intimate partner violence. Table S5 Unadjusted and adjusted odds ratio of women having 8 or more Antenatal care visits associated with women’s justification of intimate partner violence. [file 12905_2022_1780_MOESM1_ESM.docx]

**Supplementary Table 1: A summary of total households and women samples included in the study.**

| **Country** | **Total households** | **Household response rate** | **Total women of reproductive age (15-49 years)** | **Women response rate** | **Total women included in the study*** |
| --- | --- | --- | --- | --- | --- |
| Afghanistan, 2010-11 | 13116 | 98.5% | 21290 | 96.5% | 4865 |
| Bhutan, 2010 | 14676 | 98.4% | 14018 | 83.3% | 2368 |
| Nepal, 2014 | 12405 | 98.5% | 14162 | 94.8% | 2048 |
| Pakistan (Punjab), 2014 | 38405 | 98.0% | 53668 | 88.0% | 10653 |
| Pakistan (Sindh), 2014 | 17014 | 94.4% | 26647 | 89.1% | 6095 |
| **Total** | **95616** | **97.4%** | **129785** | **89.5%** | **26029** |

**Total married reproductive aged women having childbirth 2 years prior to the survey.*

**Supplementary Table 2: Definition of outcome, explanatory/exposure, and covariates included in the study.**

| **Variables** | **Variable definition and coding** |
| --- | --- |
| **Outcome variables** |  |
| Family planning methods use | Women aged 15-49 years who are currently married or in union and using (or whose partner is using) a (modern or traditional) contraceptive method; coded as 1 “using FP methods” and 0 “Not using FP methods”. |
| At least one ANC visits | Women aged 15-49 years with a live birth in the last 2 years who were at least once attended by skilled health personnel during their last pregnancy that led to a live birth; coded as 1 “had at least one ANC visit” and 0 “not having ANC visit”. |
| Four or more ANC visits | Women aged 15-49 years with a live birth in the last 2 years who were at least four times attended by skilled health personnel during their last pregnancy that led to a live birth; coded as 1 “had at least four ANC visits” and 0 “not having at least 4 ANC visits”. |
| Institutional Delivery | Women aged 15-49 years with a live birth in the last 2 years whose most recent live birth was delivered in a health-facilities; coded as 1 “having institutional delivery” and 0 “Not having institutional delivery”. |
| PNC services | Women aged 15-49 years with a live birth in the last 2 years who received a health check while in facility or at home following delivery, or a postnatal care visit within 2 days atter delivery; coded as 1 “having PNC visit” and 0 “Not having PNC visit”. |
| **Main exposure variables** | |
| Women’s justification of IPV | Women aged 15-49 years who are currently married or living together with their partners and who justified wife-beating from their partners for any of the five reasons such as going out without informing their husband, neglecting children, arguing with their husband, refusing to have sex with husband/partner and burning food. The variable was coded as 1 “Justifying wife beating for at least one of the reasons/conditions” and 0 “not justifying wife beating for any of the reasons/conditions” |
| Levels of women’s justification of IPV | Women aged 15-49 years who are currently married or living together with their partners and who justified wife-beating from their partners for a number of reasons/conditions presented to them, ranging from 0 “not justifying IPV for any reason/condition’ to 5 “justifying IPV for all five reasons/conditions”. |
| **Other covariates:** | |
| Women’s age | Women’s age categorized into seven groups (15-19 years, 20-24 years, 25-29 years, 30-34 years, 35-39 years, 40-44 years and 45-49 years) |
| Women’s education status | Women’s education status categorized into three groups (None, Primary level and Secondary level or higher) |
| Wealth quintiles | Household wealth quintiles of the women categorized into either Poorest, Poor, Middle, Rich and Richest group. |
| Area of residence | Residential location of the women categorized as either rural or urban; coded as 1 “Urban” 2 “Rural” |
| Women’s age at first marriage/union | Age of women (in years) during their first marriage/union |
| Age of husband/partner | Age of husband/partner (in years) |
| Number of children ever born | Number of children ever born (continuous variable) |

**Supplementary Table 3: Adjusted odds ratio for at least one Antenatal care visit associated with women’s justification of intimate partner violence by area of residence and its interaction.**

| **Maternal Health Care service** | **Area of residence*** | | **Interaction term**  **(aOR and *P*-value)^†^** |
| --- | --- | --- | --- |
|  | **Urban** | **Rural** |  |
| Utilizing at least one Antenatal care visit | 0.67 (0.60, 0.75) | 0.83 (0.73, 0.94) | 0.81 (95%CI: 0.93, 0.70); *P*=0.003 |

*Surveys countries and year: Afghanistan, 2010-11; Bhutan, 2010; Nepal, 2014; Pakistan (Punjab), 2014 and Pakistan (Sindh), 2014.*

**Adjusted for women’s age, women’s education, women’s age at first marriage/union, age of husband, wealth quintiles, and number of children ever born.*

**^†^***Adjusted for women’s age, women’s education, area of residence, women’s age at first marriage/union, age of husband, wealth quintiles, and number of children ever born.*

**Supplementary Table 4: Delay in timing (month) for the first Antenatal care visit associated with women’s justification of intimate partner violence.**

| **Country** | **Delay in the first Antenatal care visit (month)*** |
| --- | --- |
| Nepal | 0.06 (-0.09, 0.20) |
| Pakistan Punjab | 0.22 (0.13, 0.31) |
| Pakistan Sindh | 0.36 (0.22, 0.49) |

*Surveys countries and year: Nepal 2014; Pakistan (Punjab), 2014 and Pakistan (Sindh), 2014.*

**Adjusted for women’s age, women’s education, area of residence, women’s age at first marriage/union, age of husband, wealth quintiles, and number of children ever born.*

**Supplementary Table 5: Unadjusted and adjusted odds ratio of women having 8 or more Antenatal care visits associated with women’s justification of intimate partner violence.**

| **Country** | | **Unadjusted** | **Adjusted*** |
| --- | --- | --- | --- |
| Afghanistan | 0.22 (0.14, 0.35) | | 0.40 (0.26, 0.63) |
| Bhutan | 0.85 (0.63, 1.16) | | 0.98 (0.70, 1.37) |
| Nepal | 0.38 (0.21, 0.66) | | 0.62 (0.35, 1.10) |
| Pakistan Punjab | 0.47 (0.40, 0.54) | | 0.83 (0.71, 0.96) |
| Pakistan Sindh | 0.24 (0.19, 0.30) | | 0.74 (0.57, 0.95) |
| **All countries combined ^†^** | **0.46 (0.35, 0.59)** | | **0.76 (0.64, 0.91)** |

*Surveys countries and year: Afghanistan 2010-11; Bhutan 2010; Nepal 2014; Pakistan (Punjab), 2014 and Pakistan (Sindh), 2014.*

**Adjusted for women’s age, women’s education, area of residence, women’s age at first marriage/union, age of husband, wealth quintiles, and number of children ever born.*

**^†^***Adjusted for both cluster level women weights and country weights.*
